# High serum levels of tissue inhibitor of matrix metalloproteinase-1 during the first week of a malignant middle cerebral artery infarction in non-surviving patients

**DOI:** 10.1186/s12883-019-1401-8

**Published:** 2019-07-18

**Authors:** Leonardo Lorente, María M. Martín, Luis Ramos, Mónica Argueso, Juan J. Cáceres, Jordi Solé-Violán, Alejandro Jiménez, Juan M. Borreguero-León, Agustín F. González-Rivero, Josune Orbe, José A. Rodríguez, José A. Páramo

**Affiliations:** 1 Intensive Care Unit, Hospital Universitario de Canarias. Ofra, s/n, La Laguna, 38320 Santa Cruz de Tenerife, Spain; 2 Intensive Care Unit, Hospital Universitario Nuestra Señora de Candelaria, Crta del Rosario s/n, 38010 Santa Cruz de Tenerife, Spain; 3 Intensive Care Unit, Hospital General La Palma, Buenavista de Arriba s/n, 38713 Breña Alta, La Palma Spain; 4 Intensive Care Unit, Hospital Clínico Universitario de Valencia, Avda. Blasco Ibáñez n°17-19, 46004 Valencia, Spain; 5 Intensive Care Unit, Hospital Insular, Plaza Dr Pasteur s/n, 35016 Las Palmas de Gran Canaria, Spain; 6 Intensive Care Unit, Hospital Universitario Dr Negrín, Barranco de la Ballena s/n, 35010 Las Palmas de Gran Canaria, Spain; 7 Research Unit, Hospital Universitario de Canarias, Ofra, s/n. La Laguna, 38320 Santa Cruz de Tenerife, Spain; 8 Laboratory Department, Hospital Universitario de Canarias, Ofra, s/n. La Laguna, 38320 Santa Cruz de Tenerife, Spain; 9 Atherosclerosis Research Laboratory, CIMA-University of Navarra, Avda Pío XII n°55, 31008 Pamplona, Spain

**Keywords:** TIMP-1, Ischemic stroke, Patients, Mortality, Prognosis

## Abstract

**Background:**

Higher circulating levels of tissue inhibitor of matrix metalloproteinases (TIMP)-1 early after ischemic stroke have been associated with lower survival. The objectives of this study were to determine serum TIMP-1 levels during the first week of a severe cerebral infarction in surviving and non-surviving patients, and whether those levels during the first week could be used as a mortality biomarker for these patients.

**Methods:**

We included patients with severe malignant middle cerebral artery infarction (MMCAI) defined as computer tomography showing ischaemic changes in more than 50% of the middle cerebral artery territory and Glasgow Coma Scale (GCS) ≤ 8. We measured serum levels of matrix metalloproteinases (MMP)-9 and TIMP-1. End-point study was 30-day mortality.

**Results:**

We found higher TIMP-1 concentrations at days 1 (*p* < 0.001), 4 (*p* = 0.001), and 8 (*p* = 0.03) of MMCAI in non- urviving (*n* = 34) than in surviving (n = 34) patients. We found lower serum MMP-9 concentrations at day 1 (p = 0.03) of MMCAI and no significant differences at days 4 and 8. ROC curve analysis of TIMP-1 concentrations performed at days 1, 4, and 8 of MMCAI showed an area under curve to predict 30-day mortality of 81% (p < 0.001), 80% (p < 0.001) and 72% (*p* = 0.07) respectively.

**Conclusions:**

The new findings of our study were that non-surviving MMCAI patients showed higher serum TIMP-1 levels during the first week of MMCAI that surviving patients, and those levels during the first week of MMCAI could be used as mortality biomarkers.

## Background

Ischemic stroke produces many disabilities, deaths and healthcare expenses [1]. Matrix metalloproteinases (MMPs) are a family of proteinases whose function is to remodel the extracellular matrix (ECM) and are regulated by tissue inhibitors of matrix metalloproteinases (TIMPs). MMPs play a role in different physiological processes such as morphogenesis, tissue remodelling, menstrual cycle, and angiogenesis, and in different pathological processes such as atherosclerosis, arthritis, tumour invasion [2, 3], and cerebral ischemia [4–6].

Higher circulating MMP-9 levels have been found in ischemic stroke patients with worst neurologic outcome [7–23]. In addition, higher circulating TIMP-1 levels at day 1 of ischemic stroke have been associated with poor neurological outcome [24, 25] and lower survival [26]. However, there is no data about circulating TIMP-1 levels in surviving and non-surviving patients with cerebral infarction during the first week of cerebral infarction. Thus, the objectives of this study were to compare serum TIMP-1 levels during the first week of a severe cerebral infarction between non-surviving and surviving patients and to determine whether those levels during the first week of a severe cerebral infarction could be used as a biomarker of early mortality.

## Methods

### Design and subjects

This was an observational, prospective study, carried out in Spain in 6 Critical Care Units. The period time for patient recruitment was 2009–2012. The Institutional Review Board of the 6 hospitals approved the protocol study. The hospitals that participated in the study were: H. Insular in Las Palmas de Gran Canaria, H. General de La Palma in La Palma, H. Universitario de Canarias in La Laguna, H. Clínico Universitario de Valencia in Valencia, H. Universitario Nuestra Señora de Candelaria in Santa Cruz de Tenerife, and H. Universitario Dr. Negrín in Las Palmas de Gran Canaria. We obtained the written informed consent from some family member of the patient to participate in the study.

We included in the study patients with severe malignant middle cerebral artery infarction (MMCAI). We diagnosed ischemic stroke based on clinical and computed tomography findings [1]. We considered that patients showed a severe MMCAI when the findings of computer tomography showed ischaemic changes in more than 50% of the middle cerebral artery territory and patients showed an acute neurological deterioration consisting of a Glasgow Coma Scale (GCS) [27]≤8. We excluded those patients with age less than 18 years, inflammatory or malignant disease, or pregnancy.

We had previously determined serum levels of TIMP-1 and MMP-9 at MMCAI diagnosis in some of those patients [26]. In our current work, we have determined those levels in 68 patients on days 1, 4 and 8 of MMCAI.

### Variables recorded

We recorded the following variables from the patients: age, diabetes mellitus, sex, arterial hypertension, chronic renal failure, chronic obstructive pulmonary disease (COPD), heart failure, Acute Physiology and Chronic Health Evaluation II (APACHE II) score [28], GCS, body temperature, creatinine, sodium, bilirubin, glycaemia, lactic acid, pressure of arterial oxygen (PaO_2_), fraction inspired oxygen (FI0_2_), PaO_2_/FIO_2_ ratio, international normalized ratio (INR), fibrinogen, activated partial thromboplastin time (aPTT), leukocytes, platelets, haemoglobin, haemorrhagic transformation, infarct volume, midline shift, thrombolysis, and decompressive craniectomy. End-point study was 30-day mortality.

### Blood samples and serum level determination of MMP-9 and TIMP-1

Serum blood samples were obtained on days 1, 4 and 8 of MMCAI and frozen at − 80 °C until assay determinations in the Atherosclerosis Research Laboratory in CIMA-Navarra University (Pamplona, Spain). MMP-9 and TIMP-1 determinations were performed by ELISAs using the kits Quantikine® (R&D Systems, Abingdon, United Kingdom). The intra-assay coefficient of variation (CV) in both techniques was 5%, the inter-assay CV for each assay were 8 and 5% respectively, and detection limit were 0.16 ng/mL and 0.08 ng/mL respectively.

### Statistical methods

We tested normality assumption. Continuous variables that showed a normal distribution were reported as mean (standard deviation) and compared between patient groups (surviving and non-surviving) with Student’s t test. Continuous variables that were not normally distributed were reported as median (interquartile ranges) and compared between patient groups with Wilcoxon-Mann-Whitney test. Categorical variables were reported as frequencies (percentages) and compared between patient groups with chi-square test. We used the technique of receiver operating characteristic (ROC) to determine the capacity for 30-day mortality prediction by serum TIMP-1 levels at days 1, 4 and 8 of MMCAI. We used the Spearman correlation coefficient to test the association between continuous variables. We carried out a multiple logistic regression analysis to determine the association between serum TIMP-1 levels and 30 day-mortality controlling for lactic acid, GCS, and platelet count. We used SPSS 17.0 (SPSS Inc., Chicago, IL, USA), NCSS 2000 (Kaysville, Utah) and LogXact 4.1 (Cytel Co., Cambridge, MA) to do statistical analyses. We accepted as statistically significant those *p*-values lower than 0.05.

## Results

Table 1 shows clinical variables of surviving (*n* = 34) and non-surviving (n = 34) patients. We did not find significant differences between groups in age, diabetes mellitus, sex, arterial hypertension, chronic renal failure, COPD, heart failure, APACHE-II, body temperature, creatinine, sodium, bilirubin, glycaemia, lactic acid, PaO_2_, PaO_2_/FIO_2_ ratio, INR, fibrinogen, aPTT, leukocytes, haemoglobin, haemorrhagic transformation, infarct volume, midline shift, thrombolysis, or decompressive craniectomy. Non-surviving patients showed lower GCS and platelets than survivors.

**Table 1:** Clinical and biochemical characteristics of surviving and non-surviving MMCAI patients

	Survivors (n = 34)	Non-survivors (n = 34)	*P*- value
Age (years) - median (p 25–75)	59 (47–68)	63 (53–70)	0.36
Diabetes mellitus - n (%)	4 (11.8)	9 (26.5)	0.22
Gender female - n (%)	14 (41.2)	13 (38.2)	0.99
Arterial hypertension - n (%)	19 (55.9)	16 (47.1)	0.63
Chronic renal failure - n (%)	2 (5.9)	2 (5.9)	0.99
COPD - n (%)	1 (2.9)	1 (2.9)	0.99
Heart failure - n (%)	1 (2.9)	1 (2.9)	0.99
APACHE-II score - mean ± SD	20.38 ± 6.89	22.85 ± 5.93	0.12
GCS score - median (p 25–75)	7 (6–8)	6 (3–7)	0.01
Temperature (°C) - median (p 25–75)	36.4 (36.0–37.0)	36.9 (36.0–37.3)	0.15
Creatinine (mg/dl) - median (p 25–75)	0.80 (0.60–1.13)	1.00 (0.70–1.25)	0.19
Sodium (mEq/L)- median (p 25–75)	139 (136–145)	140 (139–145)	0.38
Bilirubin (mg/dl) - median (p 25–75)	0.60 (0.40–0.83)	0.60 (0.33–1.10)	0.95
Glycemia (g/dL) - median (p 25–75)	127 (100–170)	136 (118–162)	0.40
Lactic acid (mmol/L)-median (p 25–75)	1.20 (0.90–1.70)	1.55 (1.00–2.70)	0.05
PaO2 (mmHg) - median (p 25–75)	156 (105–293)	115 (94–267)	0.26
PaO2/FI0_2_ ratio - mean ± SD	298 ± 116	262 ± 96	0.17
INR - median (p 25–75)	1.06 (1.00–1.20)	1.20 (1.01–1.31)	0.07
Fibrinogen (mg/dl) - median (p 25–75)	443 (416–489)	419 (337–631)	0.90
aPTT (seconds) - median (p 25–75)	28 (25–30)	27 (26–32)	0.91
Leukocytes-median*10^3^/mm^3^ (p 25–75)	12.4 (9.6–16.9)	13.9 (9.7–20.1)	0.32
Hemoglobin (g/dL) - mean ± SD	12.60 ± 1.73	12.82 ± 1.96	0.67
Platelets - mean ± SD	225242 ± 84667	179382 ± 61975	0.01
Haemorrhagic transformation - n (%)	7 (20.6)	6 (17.6)	0.99
Volumen infarction (ml) - mean ± SD	162 ± 82	182 ± 110	0.61
Midline shift (mm) - mean ± SD	7.47 ± 6.14	9.09 ± 6.60	0.44
Thrombolysis - n (%)	11 (32.4)	10 (29.4)	0.99
Decompressive craniectomy – n (%)	9 (26.5)	7 (20.6)	0.78
MMP-9 (ng/mL) - median (p 25–75)	839 (719–1192)	708 (396–949)	0.03
TIMP-1 (ng/mL) - median (p 25–75)	206 (173–262)	375 (248–468)	< 0.001

*P 25–75* Percentile 25th–75th, *SD* Standard deviation, *COPD* Chronic obstructive pulmonary disease, *APACHE II* Acute Physiology and Chronic Health Evaluation, *GCS* Glasgow Coma Scale, *PaO*_*2*_ Pressure of arterial oxygen/fraction inspired oxygen, *FIO*_*2*_ Pressure of arterial oxygen/fraction inspired oxygen, *INR* International normalized ratio, *aPTT* Activated partial thromboplastin time, *MMP* Matrix metalloproteinase, *TIMP* Tissue inhibitor of matrix metalloproteinases

**Fig. 1:**
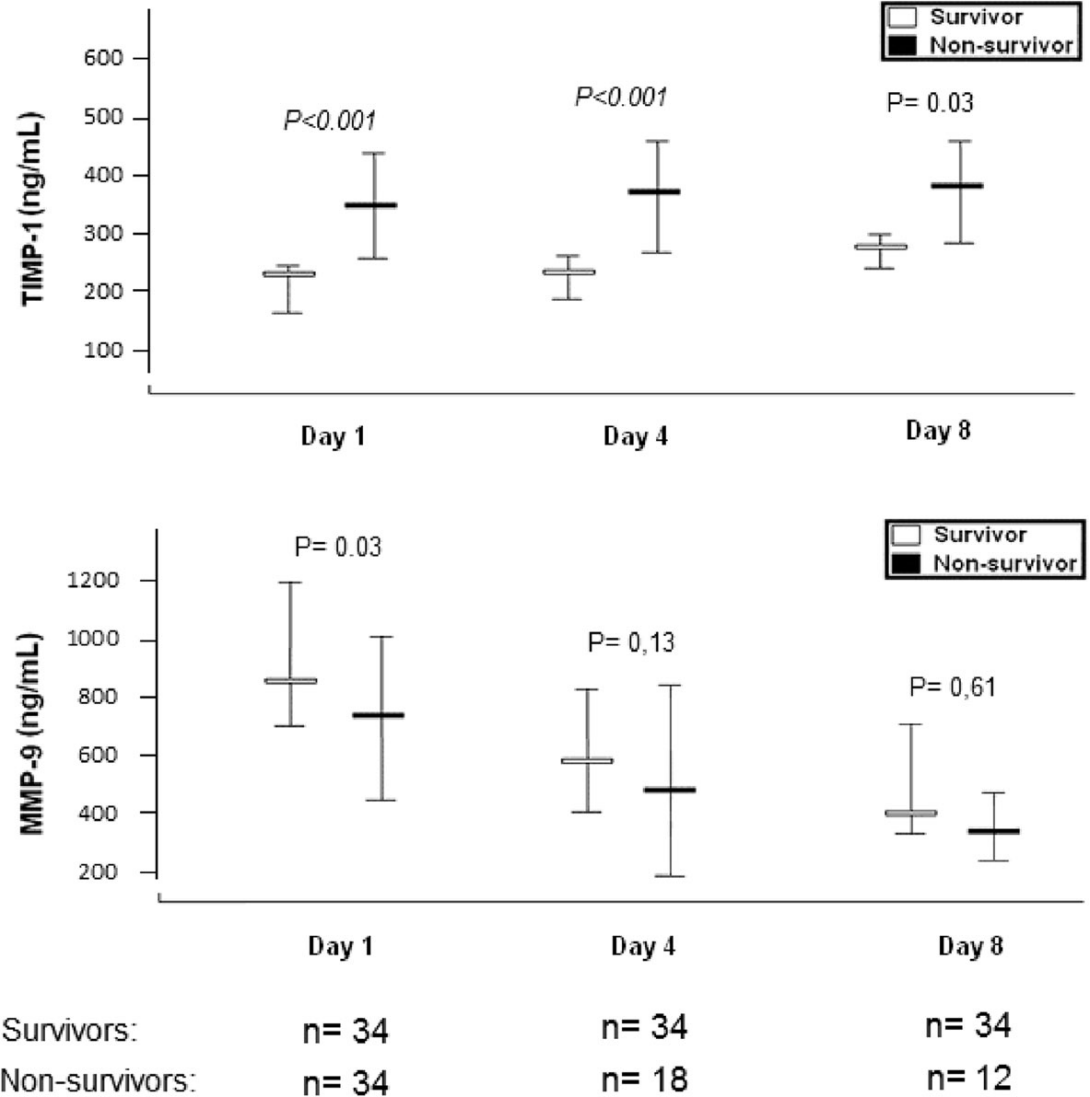
Serum TIMP-1 and MMP-9 levels at day 1, 4 and 8 of severe MMCAI in 30-day surviving and non-surviving

We found higher serum TIMP-1 concentrations at days 1 (*p* < 0.001), 4 (*p* = 0.001), and 8 (*p* = 0.03) of MMCAI in non-surviving than in the surviving patient group (Table 2 and Fig. 1). We found lower serum MMP-9 concentrations at day 1 (p = 0.03) of MMCAI in the non- surviving than in the surviving patient group; however, we did not find statistically significant differences in serum MMP-9 concentrations at days 4 and 8 (Table 2 and Fig. 1).

**Table 2:** Serum TIMP-1 and MMP-9 levels at day 1, 4 and 8 of severe MMCAI in 30-day surviving and non-surviving patients

Parameters Median and percentiles 25th -75th	Survivors	Nonsurvivors	*P*
Day 1	(n = 34)	(n = 34)	
TIMP-1 (ng/mL) - median (p 25–75)	206 (173–262)	375 (248–468)	< 0.001
MMP-9 (ng/mL) - median (p 25–75)	839 (719–1192)	708 (396–949)	0.03
Day 4	(n = 34)	(*n* = 18)	
TIMP-1 (ng/mL) - median (p 25–75)	208 (193–247)	350 (213–461)	< 0.001
MMP-9 (ng/mL) - median (p 25–75)	596 (408–792)	414 (183–884)	0.13
Day 8	(n = 34)	(*n* = 12)	
TIMP-1 (ng/mL) - median (p 25–75)	234 (213–262)	370 (211–451)	0.03
MMP-9 (ng/mL) - median (p 25–75)	370 (295–721)	361 (211–459)	0.61

**Fig. 2:**
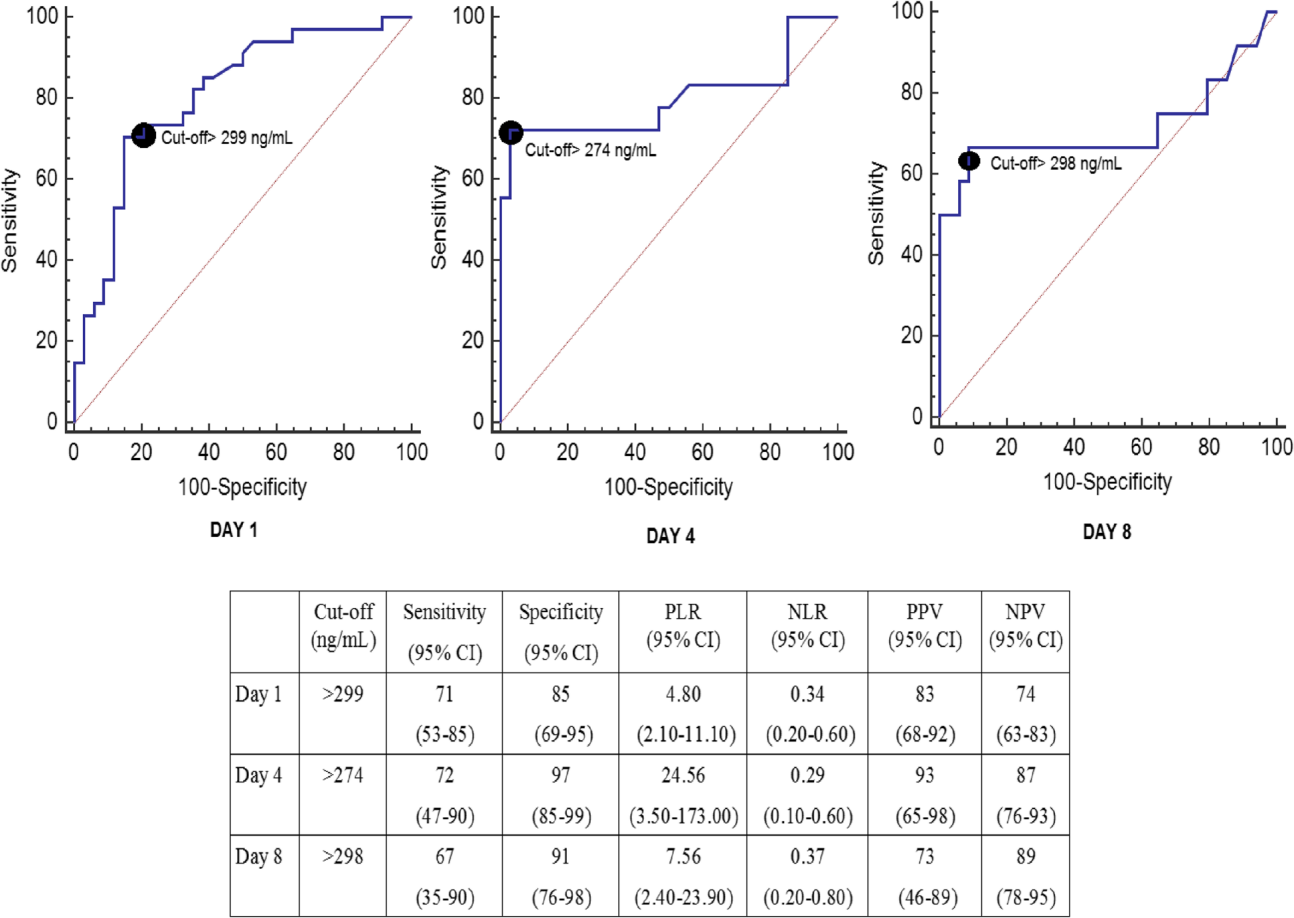
Receiver operation characteristic analysis using serum TIMP-1 levels at day 1, 4 and 8 of severe MMCAI as predictor of mortality at 30 days. CI: confidence intervals, PLR: positive likelihood ratio, NLR: negative likelihood ratio, PPV: positive predicted value, NPV: negative predicted value

ROC curve analyses assessing serum TIMP-1 concentrations at days 1, 4, and 8 of MMCAI showed an area under curve (and 95% confidence interval) to predict 30-day mortality of 81% (69–89%; p < 0.001), 80% (66–90%; p < 0.001) and 72% (56–84%, *p* = 0.07) respectively (Fig. 2).

In multiple logistic regression analysis we found that serum TIMP-1 levels were associated with 30-day mortality controlling for platelet count, GCS and lactic acid (OR = 1.011; 95% CI = 1.005–1.018; p = 0.001) (Table 3).

**Table 3:** Multiple logistic regression analysis to predict 30-day mortality

Variable	Odds Ratio	95% Confidence Interval	*P*
TIMP-1 (ng/mL)	1.011	1.005–1.018	0.001
Lactic acid (mmol/L)	1.131	0.626–2.046	0.68
Platelet count (each 1,000/mm^3^)	0.991	0.981–1.001	0.08
Glasgow Coma Scale (points)	0.707	0.497–1.005	0.054

No association between serum levels of TIMP-1 and MMP-9 at days 1 (rho = − 0.17; *p* = 0.16), 4 (rho = 0.02; *p* = 0.86) or 8 (rho = 0.11; *p* = 0.46) could be demonstrated.

## Discussion

The new findings of our study were that non-surviving MMCAI patients showed higher serum TIMP-1 levels during the first week of MMCAI than surviving patients, and that those levels could be used as mortality biomarkers.

Previous studies found higher brain concentrations of TIMP-1 in infarcted than in healthy brain areas [29], higher monocytes expression of TIMP-1 in ischemic stroke patients than in healthy subjects [30], and higher circulating levels of TIMP-1 in ischemic stroke patients than in controls [31–35]. In addition, higher circulating TIMP-1 levels at day 1 of ischemic stroke have been previously associated with poor neurological outcome [24, 25] and lower survival [26]. Thus, the finding in our current study that serum TIMP-1 levels during the first week of a severe MMCAI were higher in surviving than in non-surviving patients is a novel finding. Previously, we also found that circulating TIMP-1 levels on the first day of MMCAI could be used as biomarkers of mortality [26]. Therefore, according to the results of the ROC analyses, serum TIMP-1 levels on days 1, 4 and 8 of MMCAI could be used for 30-day mortality prediction of those patients, being this is another novel finding of our study. Patients with serum TIMP-1 levels higher than 299 ng/mL on day 1 of MMCAI, higher than 274 ng/mL on day 4 or higher than 298 ng/mL on day 8 have higher risk of death during the first 30 days than patients with lower levels.

We believe that those high TIMP-1 levels during the first week of cerebral infarction that we found in non-surviving compared to surviving patients do not contribute to the death of patients. We think that those high TIMP-1 levels in non-surviving patients could be due to the attempt to maintain the balance between the activity of MMPs and TIMPs.

We recognized the limitations of our study such as that we did not report data about others MMPs and TIMPs. In addition, we have determined serum MMP-9 levels by ELISA that detects the active form and latent pro-form; however, we have not determined the active form by gelatin zymography [36]. Besides, we determined serum TIMP-1 levels at days 1, 4, and 8 of MMCAI; however, it could have been interesting the determination of those levels at other moments of evolution as well. In addition, we have not registered all treatment for each patient; however, we did not find significant differences between surviving and non-surviving patients in thrombolysis and decompressive craniectomy.

In rat models of ischemic stroke the administration of MMP activity modulators showed beneficial effects such as to reduce blood-brain barrier leakage, infarct volume, neurological impaired and death rate [37–45].

We think that the findings of our study about the potential use of serum TIMP-1 levels during the first week of cerebral infarction for 30-day mortality prediction of those patients, and the finding in animal studies about the potential benefits of MMP activity modulators could motivate the research about the role of serum TIMP-1 levels for the mortality prediction and the MMP modulation to reduce death risk of these patients.

## Conclusions

The new findings of our study were that non-surviving MMCAI patients showed higher serum TIMP-1 levels during the first week of MMCAI that surviving patients, and that those levels during the first week of MMCAI could be used as mortality biomarkers.

## Data Availability

The datasets used and/or analysed during the current study are available from the corresponding author on reasonable request.

## Abbreviations

APACHE II: Acute Physiology and Chronic Health Evaluation
aPTT: Activated partial thromboplastin time
FIO_2_: Pressure of arterial oxygen/fraction inspired oxygen
GCS: Glasgow Coma Scale
INR: International normalized ratio
MMP: Matrix metalloproteinases
PaO_2_: Pressure of arterial oxygen/fraction inspired oxygen
TIMP: Tissue inhibitor of matrix metalloproteinases
